# Knowledge and attitude of nurses in primary health care regarding climate change in Mpumalanga

**DOI:** 10.4102/hsag.v30i0.3105

**Published:** 2025-08-28

**Authors:** Marriot M. Mabena, Ramadimetja S. Mooa, Andries Masenge, Nombulelo V. Sepeng

**Affiliations:** 1 Department of Nursing, Faculty of Health Sciences, University of Pretoria, Pretoria, South Africa; 2 Internal Statistical Consultation Service, Department of Statistics, University of Pretoria, Pretoria, South Africa

**Keywords:** attitude, climate change, knowledge, primary health care, professional nurse

## Abstract

**Background:**

Climate change is increasingly recognised as a critical public health challenge, a perspective reinforced by its inclusion as Sustainable Development Goal 13. Given its significant disease burden, climate change should be reframed as a leading health priority, demanding an immediate and coordinated response from the health sector.

**Aim:**

The aim of the study was to assess the knowledge and attitudes of professional nurses in primary health care regarding climate change.

**Setting:**

The study was conducted in Nkangala District, Mpumalanga province.

**Methods:**

A non-experimental, descriptive and quantitative research design, employing a self-administered questionnaire, was used to collect data. A comprehensive sampling of the entire population was conducted in the Thembisile Hani Local Municipality, Nkangala District, Mpumalanga, because of the small population size.

**Results:**

About 82% of the professional nurses in primary health care had knowledge regarding the effects of climate change, and 23% did not link climate change to diseases. Regarding nurses’ attitudes and perceptions, approximately 66.2% strongly agreed that understanding the issue is crucial to providing effective patient care. In support, 29.8% agreed that it is essential to understand the issue to help patients, 3.3% were neutral, while 0.7% strongly disagreed and 0% disagreed.

**Conclusion:**

The findings of the study highlighted the importance of educating registered nurses about the mental health effects of climate change. It also emphasised the need to formally include climate change in the nursing curriculum, as many nurses currently depend on informal education to understand its implications.

**Contribution:**

The results of the study will contribute new knowledge regarding climate change, its impact on health and its implications for the nursing profession. In addition, the study emphasised the importance of incorporating climate change into the nursing curriculum.

## Introduction

Climate change is increasingly recognised as a significant threat to global public health, a concern highlighted by the United Nations through Sustainable Development Goal (SDG) 13, which calls for urgent action to combat climate change and its impacts (Xiao et al. 2016). Population health is a fundamental goal for achieving sustainable development. Climate change is characterised by a change in weather conditions, including the rise in sea levels, changes in precipitation patterns, increasingly acidic oceans and the regularity and intensity of severe weather events (Romm 2022). Recently, climate change has been outlined as a health challenge. According to Xiao et al. (2016), the United Nations have endorsed this concept through SDG 13. Similar to others, this goal has endorsed actions and plans aimed at integrating preventive health measures. The endorsed actions and plans clarify how each country must pursue strategies to address the negative impact of climate change. This implies that countries have to mandate their state departments, such as health and environment, as well as parastatals, to prioritise climate change in their national plans (Morton, Pencheon & Bickler 2019).

Available literature on climate change indicates that this phenomenon is one of the most critical concerns for human health. Despite the magnitude and negative impacts of climate change on human health, a significant number of nurses appear to struggle with understanding what climate change is. Xiao et al. (2016) indicate that 76% of nurses in central China struggled to understand how climate change affects public health. It is further indicated that 24% of nurses lacked knowledge of the interaction between health and climate change, and only 39% recognised that some activities in their line of duty could help mitigate the development of climate change (Xiao et al. 2016).

Climate change contributes to infectious diseases through increased temperatures, a rise in vector-borne diseases, outbreaks of food- and water-borne diseases and, most likely, the current Listeria epidemic, as well as potential effects on mental health because of social stressors. The national analysis of mortality data across South African Districts revealed that 34% of deaths were associated with extreme temperatures, with cold and heat spells contributing to 3,4% mortality rate (Chersich et al. 2018). Similarly, Lister et al. (2022) and Yeboah, Adegboye and Kneafsey (2024) state that nurses in SA have varying levels of knowledge about climate change and its impact on health. Furthermore, Tiitta et al. (2024) report that nurses acknowledged that they have a role to play in their health promotion activities and are willing to learn about climate change, as they have encountered it through social media rather than their training curriculum.

According to Carlsen and Bruggemann (2022), the SDG report highlights that climate change is accelerating faster than anticipated, with 2019 recorded as the second warmest year on record. In relation to the health system, this was observed in various ways, particularly in diseases such as chest infections, heatstroke, malnutrition, vector-borne diseases and other communicable diseases, which are managed in different primary health care (PHC) facilities (Lorenzon et al. 2025). Bein, Karagiannidis and Quintel (2020) concur that the increasing clustering of greenhouse gases, air pollution, veld fires, lengthy heat waves, drought and floods leads to increased respiratory morbidity and mortality. Climate change also contributes to the spread of infectious diseases because of increased temperatures and heavy rainfall, leading to a rise in vector-borne diseases, such as malaria (Komen et al. 2015).

Primary health care nurses play a key role in disease prevention. Blashki, McMichael and Karoly (2007) confirm that PHC services are strategically positioned to play a crucial role in mitigating the negative impacts of climate change. Tiitta et al. (2024) reveal that the nurse’s training curriculum has limitations regarding climate change and health. Studies by Lister et al. (2022) and Yeboah et al. (2024) report similar results regarding the limited discussion of climate change related diseases in the nursing curriculum. Furthermore, these studies revealed that the limited information nurses have regarding climate change was obtained from social media. As a result, nurses struggle to confidently integrate climate change discussions into their daily health education sessions (Yeboah et al. 2024).

The 2030 Agenda for Sustainable Development, including SDG 17, establishes a framework for global progress, health and well-being. Two key instruments explicitly align with SDG 3, emphasising strategic health interventions to ensure healthy lives for all. Achieving this goal and leaving no one behind requires a strong PHC approach (World Health Organization 2019). As frontline providers at the point of entry into the health system, PHC nurses must be equipped with knowledge of climate change as a scientific inquiry.

### Problem statement

South Africa is a party to the Paris Agreement, which is part of the United Nations Framework Convention on Climate Change (Dimitrov 2016). This agreement, along with others such as the 2030 Agenda for Sustainable Development and SDG 17, mandates United Nations (UN) member states to expedite the development of strategies that will curb climate change and its adverse effects on population health by 2030 (Kumar & Ayedee 2021). Despite these protocols and commitments, there exists a disconnection between climate change and PHC in SA. It is reported that most political declarations focused on the implications of climate change for health but failed to establish clear links or provide further clarification (Kadandale et al. 2020).

To address the health impacts of climate change, SA has to prioritise it as an urgent public health issue and strengthen context-specific responses. Enhancing healthcare providers’ knowledge, particularly that of primary care nurses, is crucial to mitigating the disease burdens associated with climate change (Mabena 2024). The South African population is affected by extreme weather conditions, including heatwaves, droughts, rain-related floods and storms (Chersich et al. 2018). Extreme climatic conditions directly contribute to severe health issues, including premature deaths, cardiovascular diseases, vector-borne illnesses, food-borne infections such as Salmonella and mental health disorders such as grief and behavioural health challenges (Ugochukwu 2013). Ugochukwu (2013) further states that, indirectly, these conditions exacerbate food and water insecurity because of droughts and floods, undermining agricultural productivity and further threatening public health.

Lister et al. (2022) and Yeboah et al. (2024) found that South African nurses lack sufficient knowledge about climate change, despite their strong interest in learning more. This knowledge gap hinders the effectiveness of community health education, disease prevention and health promotion efforts. However, there is limited local research on the knowledge and attitudes of professional nurses in PHC facilities, particularly in the Nkangala District of Mpumalanga province, regarding the climate change phenomenon.

### Aim of the study

The aim of this study was to investigate the knowledge and attitudes of the professional nurses in PHC regarding climate change in the Nkangala District of Mpumalanga.

### Objectives

- To assess the level of knowledge of professional nurses in PHC regarding climate change in the Nkangala District, Mpumalanga province.
- To determine the attitude of professional nurses in PHC regarding climate change in the Nkangala District, Mpumalanga province.
- To establish the relationship between demographic variables and the knowledge and attitudes of professional nurses in PHC regarding climate change in the Nkangala District, Mpumalanga province.

## Research methods and design

After the study was approved by the Mpumalanga Ethics Committee, the researcher introduced the study to the potential participants. A non-experimental, quantitative, descriptive research design was implemented to assess the knowledge and attitudes of PHC professional nurses regarding climate change in the Nkangala district of Mpumalanga province. A semi-structured, self-administered questionnaire was used to collect data from professional nurses in PHC, who participated voluntarily.

### Rigour

#### Validity

A previously used questionnaire was adopted and used to ensure the validity of the data collection tool (Sürücü & Maslakci 2020).

#### Reliability

A pilot study was conducted with 10 participants to test whether the questionnaire measures what it is intended to (Sürücü et al. 2020). Data were captured by the researcher on an Excel spreadsheet and sent to the statistician for analysis. The pilot study participants were advised not to participate in the actual study to ensure reliability.

### Study setting

The study was conducted in a selected local municipality, Nkangala District, in Mpumalanga province, South Africa. The selected local municipality is semi-urban. It is bordered by Dr JS Moroka in the North, Elias Motsoaledi in the North-East, Steve Tshwete in the East, Emalahleni in the South and the City of Tshwane Metro in the West. The study was conducted in health facilities that render 24 h services and 8 h clinics within this semi-rural municipality. This selected local municipality is a semi-urban area comprising 57 villages. There are 21 public health facilities: seven community health centres (CHCs) operating 24 h a day and 14 clinics operating for 8 h, 5 days a week (Monday to Friday), excluding holidays. The estimated number of professional nurses in the PHC setting was 177.

### Population and sampling

In this study, the researcher targeted professional nurses working in PHC settings in Thembisile Hani municipality, including both male and female participants, with at least 1 year of experience in PHC. These nurses were selected because of their in-depth knowledge of PHC services. Given the relatively small population of interest, a total sampling method was adopted, wherein the entire eligible population was included in the study. According to Etikan, Musa and Alkassim (2016), total population sampling involves assessing every member of the defined group. Ultimately, 177 professional nurses met the inclusion criteria and consented to participate.

### Data collection instrument

The instrument used was a self-administered questionnaire adapted from previous studies (Felicilda-Reynaldo et al. 2018; Polivka, Chaudry & Mac Crawford 2012; Ryan, Dubrow & Sherman 2020). The questionnaire was divided into three sections, namely: Section A covered the demographic data of the respondents, Section B dealt with questions assessing the knowledge, understanding and perceptions of the PHC nurses regarding the effects of climate change, and Section C assessed the attitude of the PHC nurses regarding the association of climate change and health. The researcher chose a questionnaire because it is less costly, complies with anonymity and minimises bias because it is filled in the absence of the researcher (Polit & Beck 2017). The questionnaires were in English, as all the participants understood the language.

A Likert scale is a method for rating responses from participants to measure their opinions or attitudes regarding the phenomenon under study (Grove, Burns & Gray 2012). The knowledge section in the questionnaire used a two-point Likert scale with responses of ‘Yes’ and ‘No’. The attitude section employed a five-point Likert scale with responses of ‘strongly agree’, ‘agree’, ‘neutral’, ‘strongly disagree’ and ‘disagree’.

### Data collection process

After obtaining permission from the Mpumalanga Department of Health authorities, the researcher distributed questionnaires with consent forms attached for participants to read and sign before participating in the study. The researcher collected the completed questionnaires from the different health facilities. The data collection process took 3 months.

For this study, a sample of 10 respondents was selected for a pilot study to ensure reliability. The results of this pilot study revealed that Cronbach’s alpha was 0.89, indicating that the questionnaire used in this study is reliable and valid. The respondents were advised not to participate in the actual study, and the questionnaire accurately measured what it was intended to measure, making it valid and reliable for the study. No adjustment was made to the data collection tool after the pilot study. Participation was voluntary, and all ethical principles, including anonymity and privacy, were adhered to.

### Statistical analysis

The data collected were entered into Microsoft Office 2019. The IBM SPSS Statistics version 28 was used to perform the analysis. Initially, data cleaning was performed to identify anomalies in the data. Then, the following analyses were conducted: frequency table counts and percentages. A test for associations, the Pearson chi-square test, was performed. If the number of counts in a cell is less than 5, the Fisher-Freeman-Halton test is interpreted. The null hypothesis tested is that there is no association between the demographic variables and the statements or questions. The test was performed at α < 0.05; if the *p* is < 0.05, the results will be regarded as significant. Additionally, a *z*-test for proportions was performed.

### Beneficence

Polit and Beck (eds. 2019) defined beneficence as, ‘… imposing a duty on researchers to minimise harm and maximise benefits …’ In this study, the researcher ensured that participants were not exposed to harm as a result of their participation and were informed that they would not receive monetary benefits.

### Autonomy

Varkey (2021) defined autonomy as the person’s right to make informed decisions and moral choices freely. In this study, the researcher met with eligible participants and read out their informed consent forms, outlining their ethical rights, prior to conducting the study. In addition, the rights were also attached to the data collection tools for participants to read further.

### Justice

The researcher ensured that justice was served to participants at all times by protecting their rights to fair treatment and privacy (eds. Polit & Beck 2019).

### Socio-demographic characteristics of respondents

The study included 151 respondents out of a projected population of 177. Female nurses predominated (78.9%, *n* = 118), while males comprised 21.1% (*n* = 33). Most participants (80.1%) held a nursing diploma, and 19.9% had a Bachelor’s degree in nursing. Work experience in PHC varied: 12.6% had < 3 years, 16.6% had 3–5 years, 25.8% had 6–10 years, 35.1% had 10–20 years and 9.9% had > 20 years. Detailed respondent characteristics from 21 PHC facilities are presented in Table 1.

**TABLE 1:** Socio-demographic characteristics of respondents.

Characteristics	Number	Percentage
**Gender**		
Females	118	78.19
Males	33	21.90
Total	151	-
**Highest qualification**		
Diploma	121	80.00
Degree	30	19.90
**Age (years)**		
18–30	20	13.20
30–40	58	38.40
40–50	47	31.10
50–60	26	17.20
**Years of experience**		
< 3 years	19	12.50
3–5 years	25	16.60
6–10 years	39	25.80
10–20 years	53	35.10
> 20 years	15	9.90

### Assessing primary health care nurses’ knowledge of climate change-related diseases

Table 2 presents the results of the participants’ responses to questions about their knowledge regarding climate change. Approximately 80% of the respondents were aware of the effects of climate change, whereas 20% were not. Similarly, 81% of the respondents also understood the future effects of climate change, while 19% did not. Around 86% of the respondents acknowledged that flooding-related displacement is already occurring, and 85% also predicted that it will continue to happen in the next 20 years. On the other hand, 13% did not agree that flooding-related displacement is happening, and 14% of the respondents still did not anticipate flooding-related displacement in the next 20 years. The mean results of the respondents revealed that 79% acknowledged the occurrence of water- and food-borne diseases as a result of climate change. Whereas the mean result of 20.1% did not indicate agreement that food- and water-borne diseases were already occurring because of climate change. Similarly, 80% acknowledged that water- and food-borne illnesses will still occur in the next 20 years, while 19.4% did not.

**TABLE 2:** Assessing primary health care nurses’ knowledge of climate change related diseases.

Health-related impact	Has already increased due to climate change				Will increase within the next 20 years because of climate change			
	Yes		No		Yes		No	
	*n*	%	*n*	%	*n*	%	*n*	%
1. Health-related illnesses	136	90.1	15	9.9	137	90.7	14	9.3
2. Flooding-related displacement of residents	130	86.0	21	13.9	129	85.0	22	14.0
3. Vector-borne infectious diseases	115	76.2	36	23.8	121	80.1	30	19.9
4. Water-borne diseases	132	87.4	19	12.6	128	84.4	23	15.2
5. Food-borne diseases	111	73.5	40	26.5	116	76.8	35	23.2
6. Air quality-related illnesses	135	89.4	16	10.6	129	85.4	22	14.6
7. Malnutrition	104	68.9	47	31.1	112	74.2	39	25.8
8. Disruption of healthcare services during extreme weather events	103	68.0	48	31.7	114	75.0	37	25.4
9. Anxiety, depression or other mental health conditions	119	78.8	32	21.0	118	78.0	33	21.8
10. Cold-related illness	117	77.5	34	22.5	119	78.8	32	21.2
11. Other climate change-related health impacts in your community	120	79.0	31	20.5	122	80.7	29	19.0

Regarding air quality-related illnesses, 89.4% admitted that climate change contributes to illnesses related to air quality. On the contrary, 10.6% did not acknowledge that air quality-related illnesses would occur in the next 20 years. Furthermore, 76% of respondents cited that health impacts linked to climate change such as malnutrition, anxiety, depression, various mental health conditions and cold-related illnesses are already noticeable in their community.

Twenty-three percent of the respondents showed disagreement with the view that health effects related to climate change such as malnutrition, anxiety, depression, other mental health conditions and cold-related illnesses are likely to occur in their community within the next two decades. Lastly, 68% of the respondents recognised that disruptions to healthcare services during extreme weather events occur because of climate change. While 31.7% of the respondents did not link the disruption of healthcare services during extreme weather events to climate change. Similarly, 75% of the respondents reported that disruptions to healthcare services during extreme weather events are expected to persist over the next 20 years because of climate change. While 25.4% of the respondents did not agree that the disruption of healthcare services during extreme weather events will still occur as a result of climate change.

The study had three variables with significant associations: gender, age and years of experience. However, for this article, the researcher solely focused on gender as a significant variable for discussion.

### Participants’ attitudes and perceptions towards learning health issues related to climate change

Table 3 outlines the participants’ responses to questions that measured their attitudes and perceptions towards learning about climate change. Approximately 66.2% strongly agreed that understanding the issue is crucial to providing effective patient care. In support, 29.8% agreed that it is essential to understand the issue to help patients, 3.3% were neutral, while 0.7% strongly disagreed and 0% disagreed. The majority of respondents, 52.3%, strongly agreed that they feel they can learn about the issue in the classroom, and it should be reinforced in the clinical setting. In support of the strongly agreeing respondents, 40.4% agreed that they feel they can learn about the issue in the classroom and that it should be reinforced in the clinical setting. Notably, 6.6% were neutral about learning about the matter in school, and this sentiment should be reinforced in the clinical setting.

**TABLE 3:** Participants’ attitudes and perceptions towards learning health issues related to climate change in their nursing curriculum.

Attitude and perceptions	Strongly agree		Agree		Neutral		Strongly disagree		Disagree	
	*n*	%	*n*	%	*n*	%	*n*	%	*n*	%
It is important to understand the issue so as to be able to help the patients	100	66.2	45	29.8	5	3.3	1	0.7	0	-
I feel I can learn about the issue in the classroom and it should be reinforced in the clinical setting	79	52.3	61	40.4	10	6.6	1	0.7	0	-
I think there is a role for nurses in addressing this issue, but it is not my personal interest area	23	15.2	54	35.8	39	25.8	7	4.6	28	18.5
It is important to understand this issue because nurses have an important role to play in educating patients and the public about the impacts of pollution and climate change on health	90	59.6	49	32.5	8	5.3	4	2.6	0	-
Pollution and climate change should not be included in the nursing school curriculum	9	6.0	25	16.6	13	8.6	53	35.1	51	33.7

In contrast, 6.6% of the respondents strongly disagreed with learning about the matter in the classroom and believed it should be reinforced in the clinical setting. About 15.2% of respondents strongly agreed that nurses have a role to play in addressing climate change although they felt it is not their area of interest the majority of respondents, 35.8%, agreed that nurses have a role to play in addressing climate change, they felt it is not their area of interest. Even though 25.8% of the respondents were neutral about the matter, 4.6% strongly disagreed with nurses having a role to play in addressing climate change. In support of the disagreement, 18.5% also disagreed with nurses having a role to play in addressing climate change. The majority of respondents, 59.6%, strongly agreed that it is essential to understand this issue because nurses play a crucial role in educating patients and the public about the impacts of pollution and climate change on health. Similarly, 32.5% of the respondents also agreed with the statement. Notably, 5.3% of the respondents were neutral about the importance of the issue and the role nurses play in educating patients and the public about the impacts of pollution and climate change on health. In contrast, 2.6% of the respondents strongly disagreed that it is important to understand this issue because nurses play a crucial role in educating patients and the public about the impacts of pollution and climate change on health.

A small number of respondents, 6.0%, strongly disagreed that climate change should be included in the nursing school curriculum. Yet 16.6% disagreed with the fact that pollution and climate change should not be included in the nursing school curriculum. Nonetheless, 8.6% of the respondents were neutral about including pollution and climate change in the nursing school curriculum. However, 35.1% of the respondents strongly disagree with excluding climate change from the nursing school curriculum. In support, 33.7% of the respondents also disagreed with the non-inclusion of climate change in the nursing school curriculum.

#### Associations between demographic variables and knowledge and attitudes of professional nurses in primary health care regarding climate

A closer inspection of Table 4 reveals significant associations between years of experience, age, gender and climate change. A significant association was found between years of experience and climate change-related flooding displacement, with professional nurses who had 10–20 years of experience displaying more knowledge about climate change (*p* = 0.020). Regarding water-borne diseases, a correlation was also found between years of experience and the impact of climate change. Notably, professional nurses between the ages of 30 and 40 years had more knowledge (*p* = 0.014).

**TABLE 4:** Associations between demographic variables and knowledge and attitude of professional nurses in primary health care regarding climate.

Knowledge and attitude of professional nurses in PHC regarding climate	Age	Gender	Years of experience
Do you think flooding-related displacement of residents has already increased because of climate change?	0.642	0.781	0.020
Do you think water-borne diseases will increase within the next 20 years as a result of climate change?	0.851	0.411	0.014
Do you think food-borne diseases will increase within the next 20 years as a result of climate change?	0.008	1.000	0.998
Do you think other climate change-related health impacts in your community has already increased because of climate change?	0.060	0.026	0.709
I think there is a role for nurses in addressing this issue, but it is not my personal interest area.	0.007	0.404	0.370
It is important to understand this issue because nurses have an important role to play in educating patients and the public about the impacts of pollution and climate change on health.	0.430	0.002	0.701

PHC, primary health care.

Another significant association was identified between age and climate change, with the notable finding that professional nurses between 50 and 60 years acknowledged that food-borne diseases are occurring as a result of climate change (*p* = 0.008). In addition, there was another significant association between gender and climate change, where female professional nurses were found to have more knowledge than their male counterparts, with a 63.6% increase in the occurrences of climate change-related health impacts in their community (*p* = 0.026).

Furthermore, another significant association was identified between age and climate change. Professional nurses between the ages of 30 and 40 years agreed that understanding this issue is important because nurses have a role to play in educating patients and the public about the impacts of pollution and climate change on health (*p* = 0.007). Lastly, there was also a correlation between female gender and climate change (*p* = 0.002).

## Discussion

The aim of this study was to assess the knowledge and attitudes of professional nurses in PHC regarding climate change in the Nkangala District of Mpumalanga. Tiitta et al. (2024) noted that while nursing curricula cover most known diseases, they lack sufficient content on climate change-related health impacts. The findings of this study indicate that the majority of respondents were aware of climate change and its effects on health, aligning with Xiao et al. (2016). Most participants recognised that climate change has already led to an increase in health-related illnesses and anticipated a further rise over the next two decades. This is consistent with Polivka et al. (2012), who reported similar perceptions among healthcare professionals, although their study did not specifically link malnutrition to climate change. In addition, the results of this study, in line with Nigatu, Asamoah and Kloos (2014), revealed that fewer respondents identified anxiety, depression and mental health issues as potential consequences of climate change.

This study found that while most respondents had some knowledge of climate change and its health impacts, this understanding was inconsistent and largely acquired outside formal nursing education. Since climate change is not yet integrated into nursing curricula, participants primarily gathered information from informal sources, resulting in significant knowledge gaps and variations in their understanding of the subject.

To address the critical gap in climate change awareness among nurses, the South African Nursing Council (SANC) should integrate climate education into the nursing curriculum, ensuring that future nurses are equipped with the essential knowledge. Furthermore, SANC should standardise climate change competencies across nursing programmes to promote uniformity in training. The National Department of Health (DoH) must develop evidence-based protocols and guidelines to support practicing nurses in addressing climate-related health challenges. Policy developers should also incorporate climate change considerations into health policies to enhance resilience and preparedness.

These findings indicate that most respondents not only held favourable views towards addressing climate change but were also eager to learn about its impacts through formal education to inform the public better. Furthermore, the majority of the respondents recognised the important role nurses play in climate action. Therefore, integrating climate change into nursing education curricula is strongly recommended to strengthen nursing practice, policy coherence and public health responses to climate-related challenges.

This study found a significant association between females and more positive attitudes towards climate change compared to male respondents. This aligns with Bush and Clayton’s (2023) findings on the relationship between gender and climate change knowledge, further supporting a strong correlation between gender and climate change perceptions. Women are disproportionately affected by climate change, despite possessing the knowledge and skills to engage in climate adaptation efforts (Preet et al. 2010). However, gender remains underrepresented in both research and policy documents, even as its impacts on health and other sectors are increasingly recognised (Preet et al. 2010).

The findings of this study revealed a significant association between age and positive attitudes towards climate change. This aligns with prior research by Polivka et al. (2012), which demonstrated that professional nurses aged 50–59 years exhibited substantial knowledge regarding climate change. However, contrasting evidence from Buriro et al. (2018) suggests that younger nurses, particularly fresh graduates, had stronger perceptions of the health effects of climate change, with years of experience showing a significant positive association (*p < 0.05*). Further supporting the role of age, Luque-Alcaraz et al. (2024) found a highly significant link between younger age, pro-environmental behaviour and climate change understanding (*p < 0.001*). These mixed findings highlight the complex relationship between age, experience and climate-related awareness among healthcare professionals.

This study found a significant correlation between years of experience and knowledge of climate change. Nsengiyumva et al. (2020) demonstrated that greater experience in neonatology was associated with higher climate change awareness and stronger perceptions of neonatal health risks (*p = 0.05*).

### Limitations

The study primarily focused on assessing the knowledge of professional nurses in the PHC Nkangala District regarding climate change; therefore, these results cannot be generalised to other settings.

### Recommendations and strengths

The findings of this study will contribute valuable insights into the intersection of climate change and health within the nursing profession. It recommends that the SANC, as the statutory body, revise the nursing curriculum to incorporate climate education, with a specific focus on the impact of climate change on mental health. This will enhance nurses’ ability to assess and manage climate-related conditions at the PHC level. Additionally, the study suggests integrating climate-related health diseases into the National Indicator Data Set (NIDS) and PHC documentation to track incidence and prevalence. Researchers are also encouraged to explore climate health further, including assessing healthcare workers’ understanding of climate change causes, to equip them with more effective preventive strategies.

## Conclusion

This study found that respondents had diverse levels of knowledge about climate change and its health impacts. They recognised their professional responsibility in health promotion and disease prevention, particularly in addressing climate-related health risks. A key finding was that most respondents emphasised the need to integrate climate change education into nursing curricula to establish standardised knowledge on the subject. These results underscore the importance of incorporating environmental studies and climate change into nursing education to formalise and strengthen nurses’ understanding of this critical issue.

Most importantly, respondents recognised their role in mitigating the health impacts of climate change within their communities. They acknowledge that formal training on environmental and climate-related issues could enhance their ability to educate, empower and engage communities on climate change, moving beyond purely clinical interventions. In addition, they agree that health promotion and disease prevention should be integral to their professional nursing responsibilities. However, many nurses lack formal education on climate change, limiting their capacity to inform communities about its current and future health effects, as well as preventive measures.
